# Towards a CdTe Solar Cell Efficiency Promotion: The Role of ZnO:Al and CuSCN Nanolayers

**DOI:** 10.3390/nano13081335

**Published:** 2023-04-11

**Authors:** Isaac Montoya De Los Santos, Alan A. Pérez-Orozco, Diego A. Liña-Martínez, Maykel Courel, Carlos A. Meza-Avendaño, Jorge A. Borrego-Pérez, Laura M. Pérez, David Laroze

**Affiliations:** 1 Instituto de Estudios de la Energía, Universidad del Istmo, Oaxaca 70760, Mexico; 2 Centro Universitario de los Valles, Universidad de Guadalajara, Ameca 46600, Mexico; maykelcourel@gmail.com; 3 Instituto de Investigación e Innovación en Energías Renovables, Universidad de Ciencias y Artes de Chiapas, Tuxtla Gutiérrez 29039, Mexico; carlos.meza@unicach.mx; 4 Departamento de Materiales, Facultad de Ingeniería Civil, Universidad Michoacana de San Nicolás de Hidalgo, Morelia 58004, Mexico; jorge.borrego@umich.mx; 5 Departamento de Física, FACI, Universidad de Tarapacá, Casilla 7D, Arica 1000000, Chile; lperez@uta.cl; 6 Instituto de Alta Investigación, CEDENNA, Universidad de Tarapacá, Casilla 7D, Arica 1000000, Chile; dlarozen@uta.cl

**Keywords:** CdTe solar cell, simulation, SCAPS, ZnO:Al nanolayer, CuSCN nanolayer

## Abstract

A numerical simulation is a valuable tool since it allows the optimization of both time and the cost of experimental processes for time optimization and the cost of experimental processes. In addition, it will enable the interpretation of developed measurements in complex structures, the design and optimization of solar cells, and the prediction of the optimal parameters that contribute to manufacturing a device with the best performance. In this sense, a detailed simulation study was carried out in this work by the Solar Cell Capacitance Simulator (SCAPS). In particular, we evaluate the influence of absorber and buffer thickness, absorber defect density, work function in back contact, R_s_, R_sh_, and carrier concentration on a CdTe/CdS cell to maximize its performance. Furthermore, the incorporation effect of ZnO:Al (TCO) and CuSCN (HTL) nanolayers was studied for the first time. As a result, the efficiency of the solar cell was maximized from 16.04% to 17.74% by increasing the J_sc_ and V_oc_. This work will play an essential role in enhancing the performance of CdTe-based devices with the best performance.

## 1. Introduction

Crystalline silicon is considered the leading technology in the PV market. Still, the consumption of the material and the high manufacturing costs of these cells have prompted the search for polycrystalline absorber layers of thin films that can adequately replace silicon. One material of interest is CdTe, which has the highest efficiency in thin-film solar cells, this value being above the one reported for CIGS [1]. CdTe is a semiconductor that possesses a direct optical bandgap (1.45 eV) and a high absorption coefficient (10^5^/cm) [2], which enable high quantum yields over a wide wavelength range (from ultraviolet to 827 nm), thus fitting almost optimally to the solar spectrum for photovoltaic energy conversion. In the mid-20th century, the first devices were manufactured by the evaporation of n-type CdS films on single-crystalline p-type CdTe, obtaining an efficiency of less than 5% [3]. Although 22.1% efficiency was eventually achieved, the maximum theoretical efficiency of ~29% has yet to be reached, which shows that there are still some challenges to overcome [4,5,6,7].

On the other hand, solar cell performance greatly depends on the energy barrier at the absorber/contact interface. Therefore, to reduce this barrier, a back contact with a low resistance and high work function (WF) is necessary. In recent years, many researchers have studied diverse materials employed as back contact layers for CdTe devices [8,9,10]. Some materials with high WF, such as WO_3_ [11], V_2_O_5_ [12], NiO [13], and MoO_x_ [14], are used as HTLs (hole transport layer) in these solar cells to improve their performance. On the other hand, inorganic materials can satisfy these properties, such as CuSCN, which has a WF of 5.3 eV and an optical bandgap of 3.6 eV [15]. These properties would allow CuSCN to act as an electron reflector at the back contact, which should help to repulse photogenerated electrons and reduce non-radiative recombination, improving V_oc_ [16]. Moreover, this compound can be synthesized using a low-cost solution [17,18].

On the other side, a transparent electrode (TCO) is used as a front contact to extract the photogenerated electrons to the external charge, usually indium tin oxide (ITO) or fluorine-doped tin oxide (FTO). Nevertheless, both materials possess a high elaboration cost, low electron mobility, and transmittance of around 80% [19,20]. So, it is imperative to search for materials that meet similar characteristics. In this sense, the ZnO:Al compound becomes the ideal candidate since it has a wide bandgap, excellent properties (optical and electrical), and great abundance [21,22,23]. On the contrary, both materials (SnO_2_:F and ZnO:Al) present a good alignment of the conduction band (CB) with the CdS material and a large valence band (VB). Therefore, minimal recombination is expected at the interface, as electrons pass through smoothly and holes are blocked. However, ZnO:Al has a lower refraction index (of around 7–9%) than SnO_2_:F. Then, this quality could make it more suitable for CdS/CdTe devices because it allows for promoting more significant photon absorption in the CdTe (absorber) layer [24,25,26].

Another essential requirement for a material to be used as front contact is its lattice matching with the buffer layer, so structural parameters should be considered during their coupling. Lattice-matched materials can be grown on top of one another without forming a high density of nonradiative recombination. However, when the growth of lattice-mismatched materials is attempted, many defects result from the relaxation of strain in the crystal structure [27,28]. In this sense, the ZnO:Al (hexagonal; a = b = 3.24 Å; c = 5.2 Å) [24] could fulfill this condition since there is a minor difference between its lattice constant with the CdS (hexagonal; a = b = 4.13 Å; c = 6.77 Å) [24] compared with the SnO_2_:F (tetragonal; a = b = 4.74 Å; c = 3.18 Å) [29].

Due to the complexity of solar cells, it is crucial to understand the physics behind these technologies. A helpful tool is a numerical simulation, which allows us to solve the most critical differential equations of solar cells and design and optimize advanced cell structures. SCAPS-1D is a solar cell simulation software conceived at the University of Gent, Belgium. This program was mainly designed for the CdTe and CuInSe_2_ materials. However, several extensions have improved their application capabilities to Si and GaAs solar cells and other materials, such as perovskite [30,31]. In addition, DFT has also studied similar nanolayer systems, including a variety of semiconductor heterostructures in order to analyze the bonding interfaces [32,33].

The present investigation yielded an extensive study about the impact of different parameters on the CdTe/CdS device structure by SCAPS-1D. Then, a new configuration proposal (CuSCN/CdTe/CdS/ZnO:Al) was studied for improving the device performance.

## 2. Theoretical Details

### 2.1. Device Architecture

**Figure 1:**
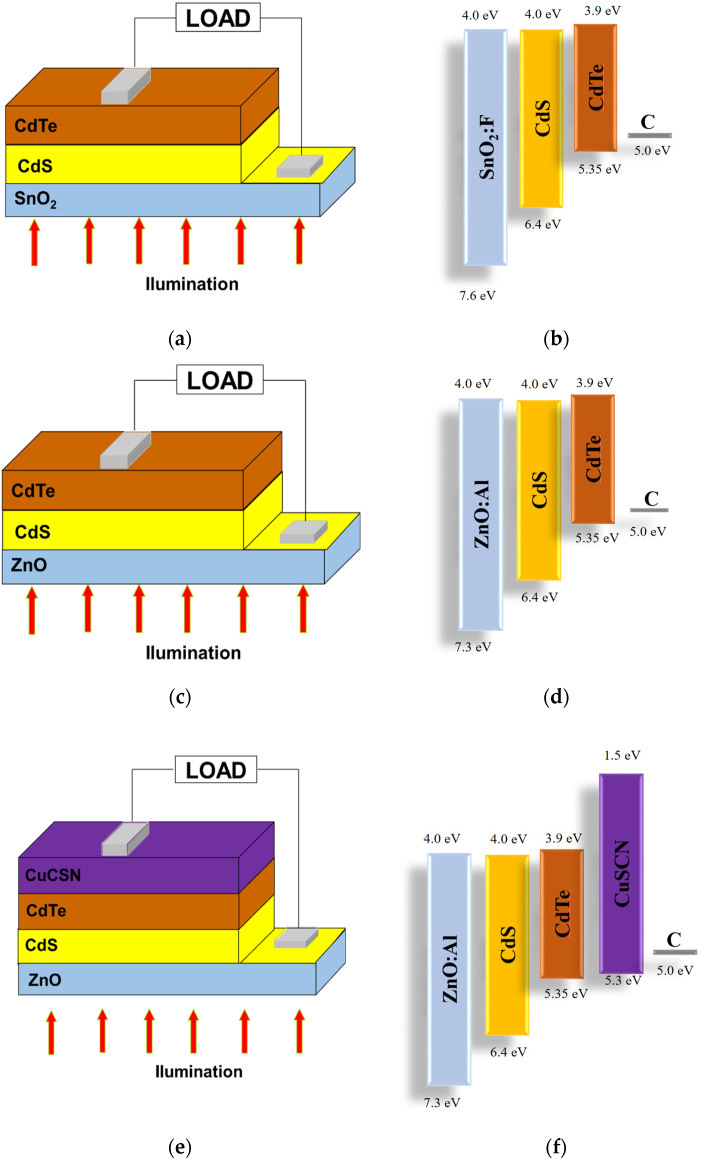
Different configurations and energy levels of CdTe solar cells. (**a**,**b**) C/CdTe/Cds/SnO_2_:F; (**c**,**d**) C/CdTe/CdS/ZnO:Al; and (**e**,**f**) C/CuSCN/CdTe/CdS/ZnO:Al.

This work investigates a CdTe solar cell in superstrate structures to analyze the impact of different parameters on the device’s performance. The reference solar cell configuration in the simulation was as follows: SnO_2_:F/CdS/CdTe/Carbon [34]. The device structures and energy levels of CdTe solar cells simulated in this work are presented below for each configuration in Figure 1. Figure 1a,b show the C/CdTe/Cds/SnO_2_:F configuration. Figure 1c,d present the C/CdTe/CdS/ZnO:Al configuration, and Figure 1e,f illustrate the C/CuSCN/CdTe/CdS/ZnO:Al configuration, which is the original proposal in this work. Here, the CuSCN unites the hole transport layer and electron-reflecting dual-layer role based on the bandgap edge offset regarding that of CdTe.

### 2.2. Simulation Parameters

The SCAPS software solves the fundamental semiconductor equations, the Poisson, the continuity equations for electrons and holes, and the drift equation [35]. The software has been used in theoretical studies due to its versatility. All material properties employed (input parameters and CdS/CdTe interface defects) are recapitulated in Table 1 and Table 2, respectively. The material parameters were chosen based on previously reported values [36,37,38,39,40,41,42,43]. Moreover, the simulations were performed under standard conditions.

## 3. Results and Discussion

### 3.1. Simulation of CdTe Base Solar Cell

**Figure 2:**
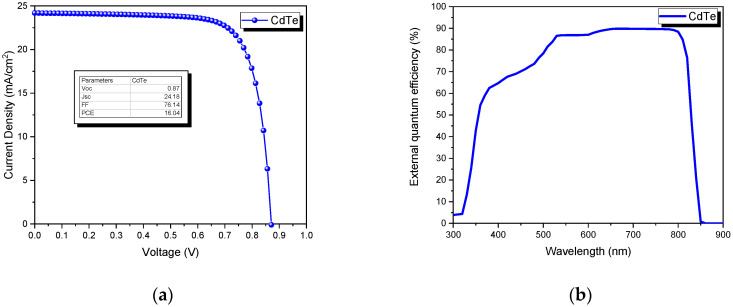
Base solar cell (C/CdTe/CdS/SnO_2_): (**a**) J-V curve, (**b**) the external quantum efficiency.

To validate our model, it was necessary to simulate a base solar cell with the typical configuration of CdTe. In this sense, the J-V and EQE curves were calculated by SCAPS and are presented in Figure 2. The output parameters are shown in the inset of Figure 2a, where a power conversion efficiency of 16.04% was obtained.

### 3.2. Effect of Thickness on the CdTe Absorber Layer

**Figure 3:**
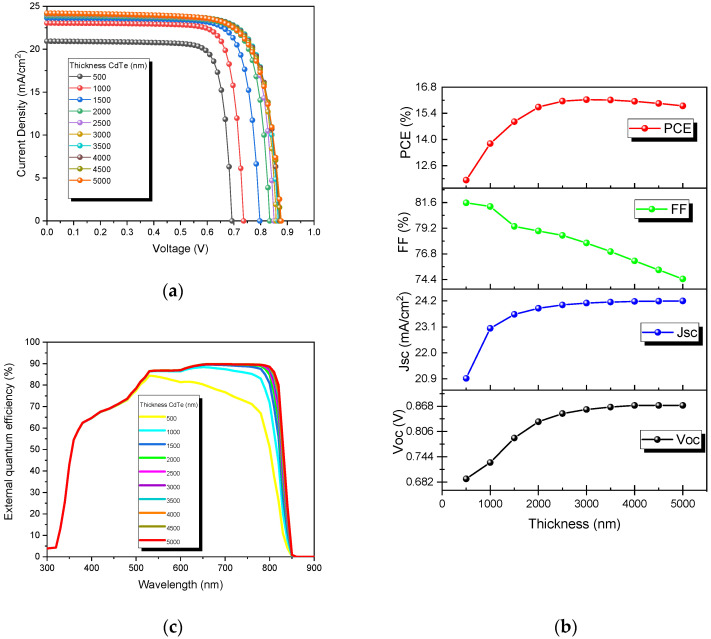
CdTe solar cells at different absorber thicknesses: (**a**) J-V curves, (**b**) output parameters, and (**c**) external quantum efficiencies.

The film thickness is a critical manufacturing parameter affecting the device’s cost and environmental protection properties. Figure 3a shows the behavior of the J-V curves of the devices as a function of the thickness of the absorber material (CdTe) while the thickness of the buffer layer (CdS) was kept constant. The simulations were carried out from 500 nm to 5000 nm. Moreover, a study of the absorber thickness on the output parameters and the external quantum efficiency is shown in Figure 3b,c, respectively.

An improvement at thicker absorber layers is observed in the J-V curves of CdTe devices (Figure 3a). The best performance was obtained at 3000 nm, as seen in Figure 3b. This result implies that the electron diffusion length would be 3 µm, which corresponds well with a Morales–Acevedo report [42]. However, some reports have demonstrated that because of the quality of the deposition of the CdTe layers, it is possible to obtain results between 1.5 and 7.5 µm [43,44]. Similarly, Table 3 shows the output parameters obtained in the simulation by varying the thickness. An increase is observed from 500 nm to 3000 nm (11.83% to 16.13%, respectively).

Results show that when the CdTe thickness was increased up to 3000 nm, the Jsc increased, improving the PCE. This is because a larger CdTe layer can absorb more photons of higher wavelengths, increasing the number of charge carriers generated in it. An improvement was observed in almost all the output parameters with the increase in thickness. However, this effect on the FF was not perceived at thicknesses greater than 3000 nm, which could be attributed to a possible increase in Rs. The plotted graph of EQE vs. wavelength for different thicknesses of the CdTe layer is presented in Figure 3c. This result shows the excellent correspondence of the absorption (855 nm) and the CdTe band gap (1.45 eV). From here, we can see that the EQE performance increased at wavelength values from about 530 nm to 850 nm as the thickness of the CdTe absorber layer increased. Still, the rate of growing quantum efficiency could be more capable for layer thickness over 3000 nm (due to there being only a slight increase at greater wavelength), which means that it was sufficient to absorb most of the incident photons. Then, considerably better photon absorption was obtained at a thicker absorber layer, which corresponds well with the J_sc_ results presented in Figure 3b. Nevertheless, we can assume that other effects were impairing the solar cell performance.

### 3.3. Effect of Thickness on the CdS Buffer Layer

**Figure 4:**
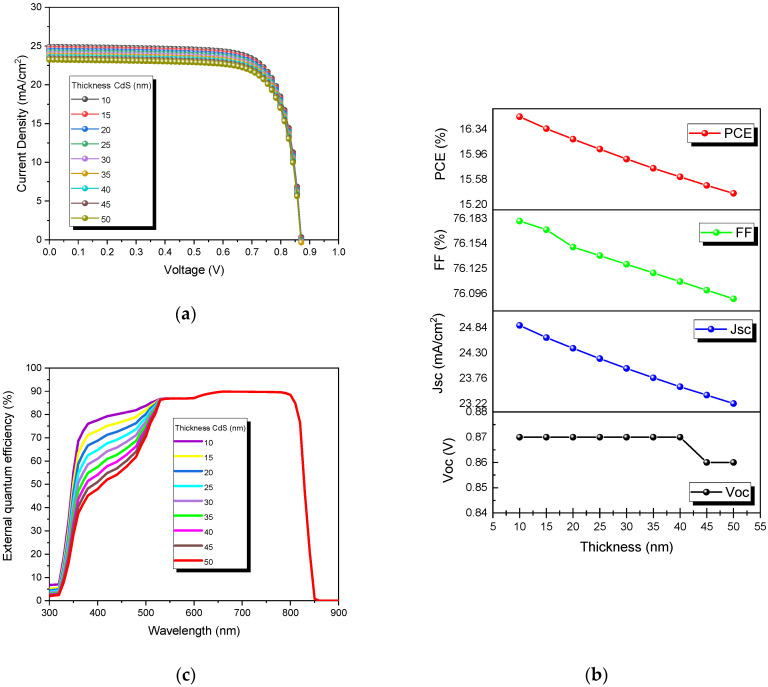
CdTe solar cells at different CdS thicknesses: (**a**) J-V curves, (**b**) output parameters, and (**c**) external quantum efficiencies.

The study of the influence of CdS thickness on the solar cell is essential to improve performance. In this sense, Figure 4a shows the behavior of the J-V curves of the devices as a function of the thickness of the buffer layer (CdS), while the CdTe layer thickness was kept constant. The simulations were carried out from 10 nm to 50 nm. The same effect and conditions were studied for the output parameters and are presented in Figure 4b. Besides, the external quantum efficiency at different thicknesses is shown in Figure 4c.

A linear decrease was observed in almost all the output parameters by the thickness increase (Figure 4a). However, this parameter (thickness) does not significantly impact the V_oc_. Table 4 shows the output parameters obtained in the simulation by varying the CdS thicknesses from 10 nm to 50 nm. It shows a decrease in all parameters at thicker CdS layers. Additionally, this behavior effect is presented in Figure 3b, where a reduction of all the output parameters can be observed as follows: V_oc_ from 0.87 V to 0.86 V, J_sc_ from 24.89 mA/cm^2^ to 23.22 mA/cm^2^, FF from 76.18% to 76.09%, and PCE from 16.53% to 15.37%.

Figure 4c shows the influence of CdS thickness on the external quantum efficiency. As can be seen, the absorption increases at a thinner CdS layer. This effect starts around 500 nm towards lower wavelengths, which corresponds well with the CdS band gap (2.4 eV). In addition, the quantum efficiency increases as the CdS thickness decreases, which indicates less parasitic absorption, making it possible to generate a more significant number of charge carriers in the absorber. This agrees with the J_sc_ (24.86 mA/cm^2^ at 10 nm) presented in Table 4.

### 3.4. Effect of Work Function in Back Contact

**Figure 5:**
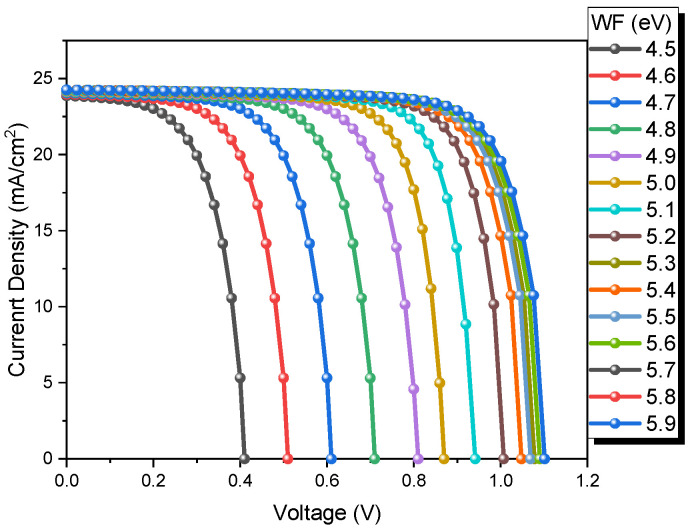
J-V curves of CdTe solar cells at different back work functions.

The energy needed to remove an electron of the highest level of the stationary Fermi distribution of a solid is known as the work function. The contacts greatly influence the performance and efficiency of solar cells because these devices are highly dependent on the front and back contact work function [45]. Figure 5 shows the J-V curves of CdTe solar cells using different work function values. In this work, an evaluation of its impact on the CdTe device performance is shown in Figure 5 (from 4.5 to 5.5 eV). An increase in V_oc_ (from 0.41 V to 1.10 V), FF (from 60.6% to 77.52%), and PCE (from 5.99% to 20.12%) is observed with the increase in work function values, while the Jsc remains the same. Therefore, materials with back contact values greater than 5.7 eV are unnecessary.

### 3.5. Effect of Defect Density (cm^−3^)

Charge carrier generation and recombination are decisive processes for the performance of CdTe solar cells. The first occurs when sunlight strikes the device, allowing the generation of photo-generated carriers in the absorber layer. The charge carriers are collected and transferred to the external current. However, many of these are lost due to the inadequate quality of the material. Higher defect density, commonly found in low-quality materials, increases the recombination rate, reducing the diffusion length of charge carriers and, thus, their lifetime. To determine the effect of defect density on CdTe solar cells’ performance, it is important to understand the recombination mechanisms (non-radiative or Shockley–Red–Hall recombination) [46].

**Figure 6:**
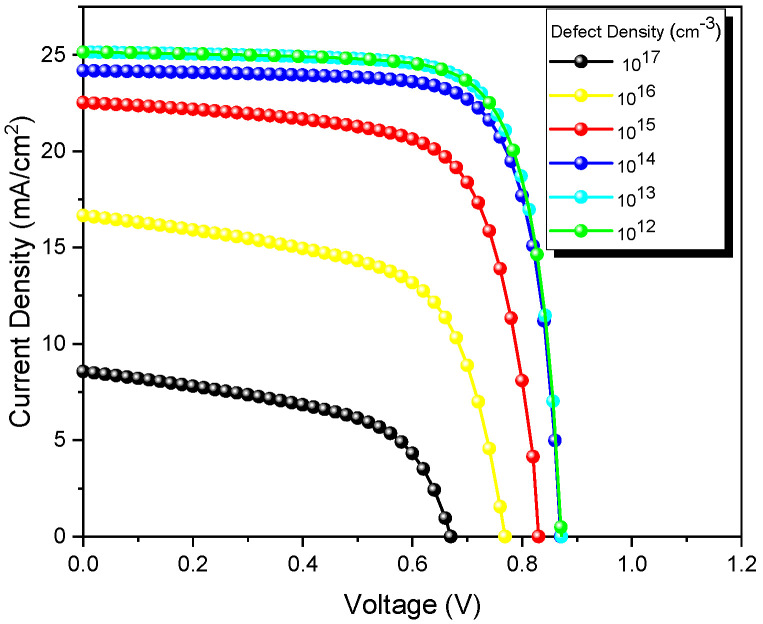
J-V curves of CdTe solar cells at different bulk defect densities of the CdTe layer.

As mentioned above, defects in absorber solar cells have a significant impact on efficiency. In this study, numerical simulations were conducted to investigate the influence of bulk defect density (cm^−3^) in the CdTe. These simulations were performed at different defect densities from 10^12^ to 10^17^ cm^−3^ (Figure 6). When analyzing the results obtained from the study of CdTe solar cells, all values (J_sc_, V_oc_, and FF) increased as the defect density of the CdTe decreased. As seen in Figure 6, the performance parameters were approximately saturated when the defect densities of the CdTe films decreased to 10^13^ cm^−3^, which reveals that an efficiency of 17.56% could be achieved only in CdTe films with remarkably low defect densities.

### 3.6. Effect of Rs and Rsh on the CdTe Solar Cell Performance

**Figure 7:**
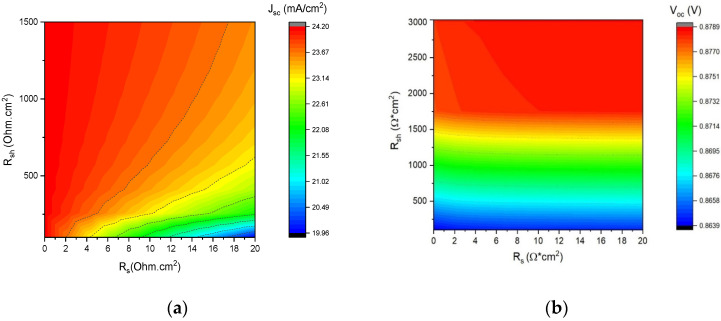

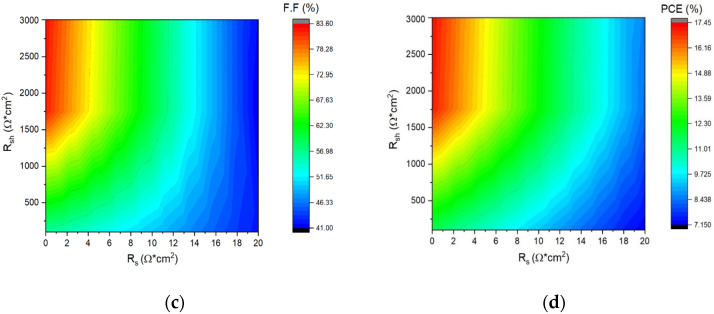
Effect of Rs and Rsh on CdTe solar cell performance: (**a**) Jsc, (**b**) V_oc_, (**c**) FF, and (**d**) PCE.

Parasitic resistances have a crucial role in a photovoltaic (PV) system. Series resistance (Rs) is generated by different bulk resistances, such as those of the semiconductor material, metallic material, and contact in the metal–semiconductor [43]. On the other hand, the shunt resistance (Rsh) is caused by leakage across the p-n junction in the vicinity of the device edge and non-peripheral regions due to crystal defects and impurity residues in the solar cell [47,48]. The effect of shunt resistance and series resistance on solar cells performance was analyzed from 0 to 20 Ω cm^2^ (Rs) and from 0 to 3000 Ω cm^2^ (Rsh) (Figure 7). A significant impact of Rs on fill factor can be observed in Figure 7. Excessively high values (around 16 Ω cm^2^) can also reduce the J_sc_. On the contrary, the V_oc_ remained the same for values of Rs but increased with higher Rsh. Also, as expected, the FF was mainly affected by the Rs, which directly affected the efficiency. Therefore, we need devices with low or negligible series resistances and very high shunt resistances to obtain high efficiencies.

### 3.7. Effect of Carrier Concentration

**Figure 8:**
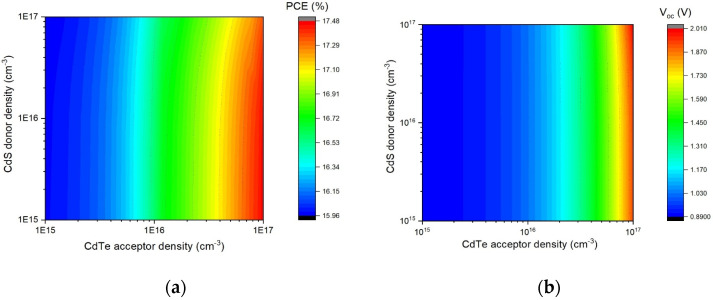
Effect of carrier concentration on CdTe solar cell performance: (**a**) PCE and (**b**) V_oc_.

Heterojunction solar cells are characterized by having two or more molecular species acting as electron donor material and electron acceptor material. The doping density of the hole generates the probability that the Fermi distribution value fills the states at the acceptor state. On the other hand, donor density is directly related to electron mobility [49,50,51,52]. Thus, to analyze the effect on the device performance, the carrier concentrations were varied from 1 × 10^15^ to 1 × 10^17^ cm^−3^ for CdS (donor densities) and CdTe (acceptor densities), respectively. As can be seen, the main impact was at greater values of acceptor concentration nearby 10^T/^cm^−3^, where the utmost efficiency of 17.48% and a V_oc_ of 2.0 V were determined (Figure 8). This effect is because the increase in the acceptor carrier concentration also resulted in the reduction of the device saturation current, and resultantly, V_oc_ increased. It is clear that the PCE and V_oc_ enlarged with the increase in the acceptor density. Indeed, hole concentration has been pointed out as the limiting factor for interface recombination in the device. On the contrary, this result shows that charge concentration in the buffer layer did not affect the performance of the photovoltaic device.

### 3.8. Enhancement of the CdTe Solar Cell Performance by Incorporating ZnO:Al and CuSCN Nanolayers

The back contact is one of the challenges of CdTe solar cell fabrication. Due to the high ionization potential of CdTe, a metal with a high WF is necessary to create an ohmic contact. Unfortunately, most metals do not fulfill this characteristic. Although there are some with a high WF, such as platinum or nickel, they are inappropriate because they tend to react with tellurium and generate disfavored phases [52]. On the contrary, metals with a low WF create a Schottky junction. This rectifying contact causes a barrier potential that can reduce FF values with increased barrier height [9]. Remarkable research is required to conceive a path to create an ohmic contact between CdTe-metal.

**Figure 9:**
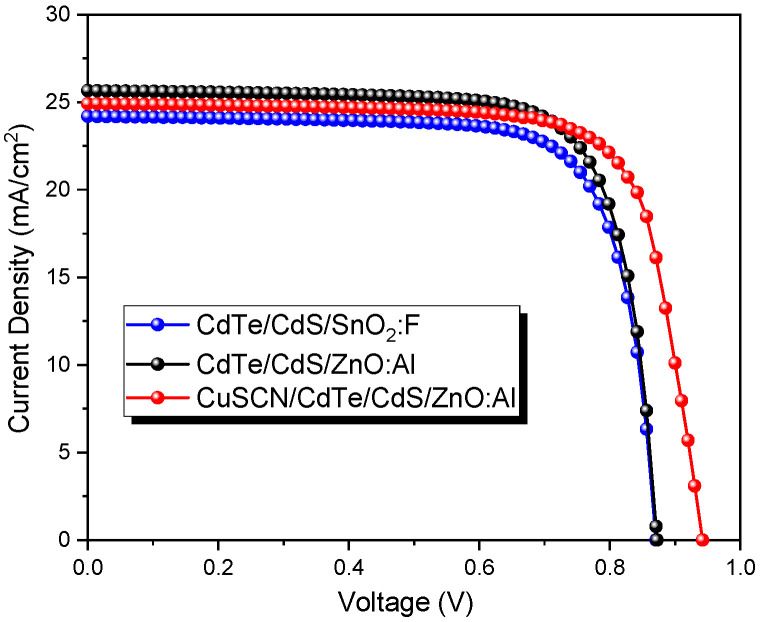
J-V curves of CdTe solar cells with different configurations.

Using Cu as a p-type dopant at the back contact has been the most productive approach, yielding hole densities of about 10^15^ cm^−2^. In this sense, researchers have reported on the use of a CuSCN layer as a hole transport layer or back contact in CdTe solar cells, i.e., HTL/back contact (CuSNC/Au) to replace the common back contact (Cu/Au) [53,54]. In addition, the TCO is located at the bottom of the solar cell, where SnO_2_:F, as mentioned in the introduction section, is commonly used. However, it is necessary to emphasize the characteristics required of a front contact (TCO) for its application in CdTe/CdS solar cells, such as high transparency (>85%), a low resistivity on the order of 10^4^ Ω cm [55,56], good stability (no degradation) to the growth of the CdS buffer layer (about 60 °C by chemical bath). In this sense, the choice of the type of TCO will depend on the type of solar cell configuration, whether they are SnO_2_:F (superstrate type) which needs a deposit temperature of around 450 °C or ZnO:Al (substrate type) that has the advantage of being deposited at room temperature [57]. Therefore, this work presents three types of CdTe solar cells. First is the standard CdS/CdTe configuration (previously studied), named cell 1. Second is the heterojunction where the ZnO:Al is used as front contact, replacing the SnO_2_:F due to its greater transparency (cell 2). And the third one, where the CuSCN material works as HTL (cell 3). The J-V curves with different configurations are shown in Figure 9.

The typical Cu/Au back contact becomes problematic due to Cu diffusion during device production conducting to degradation and compensation via extra Cu diffusion [56]. For this reason, a thin film of CuSCN (30 nm) was considered for this simulation, as was successfully experimentally reported by Paudel et al. and Montgomery et al. [53,54]. Figure 9 shows that the V_oc_ significantly increased by adding the CuSCN layer but slightly decreased in FF. This behavior is because of its high conduction band since the CuSCN operates as an electron reflector. Additionally, the CuSCN and CdTe conductivity (p-type) caused the valence band offset (CuSCN/CdTe) to be negligible. Furthermore, the CuSCN layer became a Cu source for CdTe doping (increasing hole density), which was necessary to improve the V_oc_ in CdTe devices [58]. Thus, increasing the hole concentration in the CdTe absorber can significantly reduce the back-contact barrier height and assure carrier extraction [53,54].

**Figure 10:**
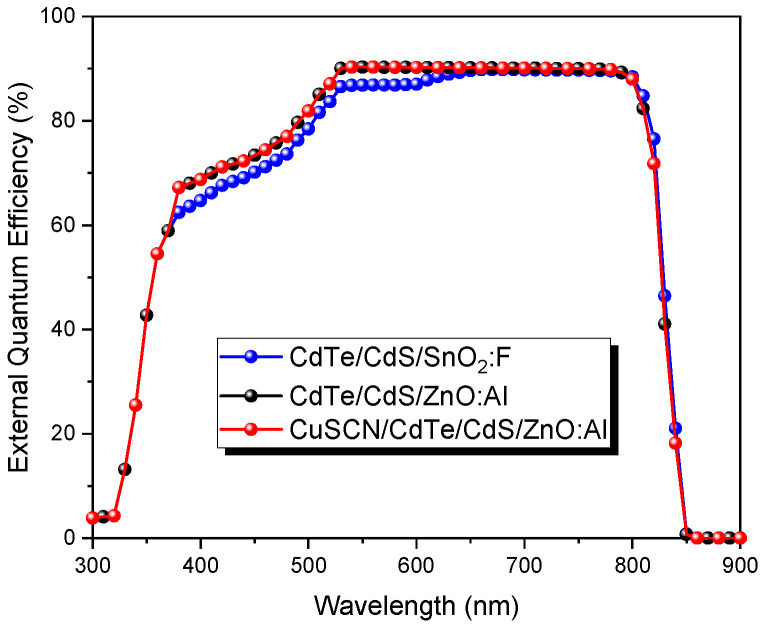
The external quantum efficiency of CdTe solar cells with different configurations.

The output parameters of all CdTe devices are presented in Table 5. It can be observed that a CuSCN layer can be applied as a back contact buffer to improve the V_oc_ from 0.87 V to 0.94 V. Results by using CuSCN as HTM are in good agreement with some reports where a significant V_oc_ (1 V) was reached [59]. Figure 10 presents the external quantum efficiency of all CdTe devices. As can be seen, a higher absorption was obtained for cell two at a wavelength between 350 and 600 nm, which corresponds with the Jsc presented in Table 5. This improvement could be related to the higher transparency of ZnO, about 90%, compared to that of SnO_2_:F (80%). Nevertheless, there was no noticeable improvement in cell 3 with the CuSCN layer.

#### 3.8.1. Effect of R_s_ in CdTe Devices with Different Configurations

**Figure 11:**
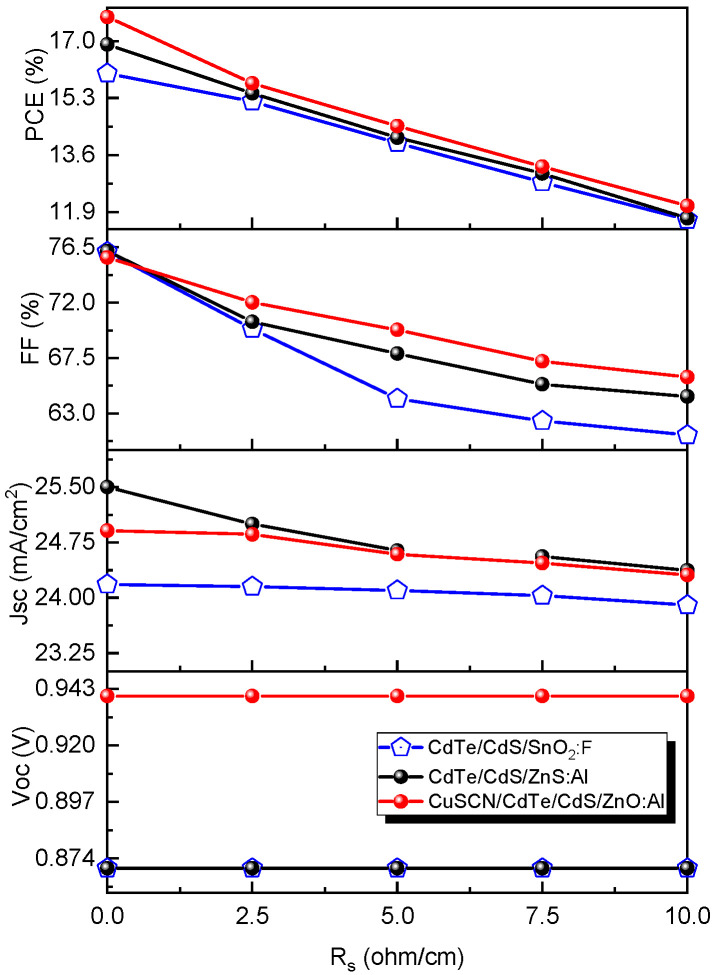
Effect of series resistance (R_s_) on the CdTe solar cells.

The Rs predominantly result from the available connections or the circuit resistance across the contacts. It is desirable to guarantee a low or negligible Rs to ensure high-efficiency solar cells. However, it is still inconceivable to achieve a perfect FF because of the deficiency of the diode. The Rs were varied from 0 to 10 Ω cm^2^ to analyze their impact on the solar cell performance using the following configurations: CdTe/CdS/SnO_2_:F (cell 1), CdTe/CdS/ZnO:Al (cell 2), and CuSCN/CdTe/CdS/CdS/ZnO:Al (cell 3). Figure 11 presents the impact of Rs on the solar cell parameters. These results illustrate that all solar cell structures were negatively affected by increasing the series resistance, as demonstrated by the sharp decrease in efficiency with rising Rs.

In all configurations, the V_oc_ remained constant with increasing Rs values. As this value increased from 0 Ω cm^2^ to 10 Ω cm^2^, the efficiency decreased from 16.04% to 11.67% (Cell 1), from 16.90% to 11.72% (Cell 2), and from 17.72% to 12.09% (Cell 3). Nevertheless, better performance was shown for the device with the CuSCN layer (compared with the other devices). The effect of adding the CuSCN and ZnO:Al nanolayers in the device design is revealed in Figure 11. As a result, a minor detrimental impact on all output parameters was observed by the increase in Rs, which can be attributed to a combined effect of these layers on the solar cell performance. This means that for superior transmittance by the ZnO:Al layer, better photogeneration is achieved. Additionally, in the case of the nanolayer of CuSCN, there are two contributions: first, it usually forms an electron reflecting barrier, which helps to reduce leakage currents; and second, it improves the extraction of carriers (holes) to the back contact.

#### 3.8.2. Optimization of the CdTe Solar Cell

**Figure 12:**
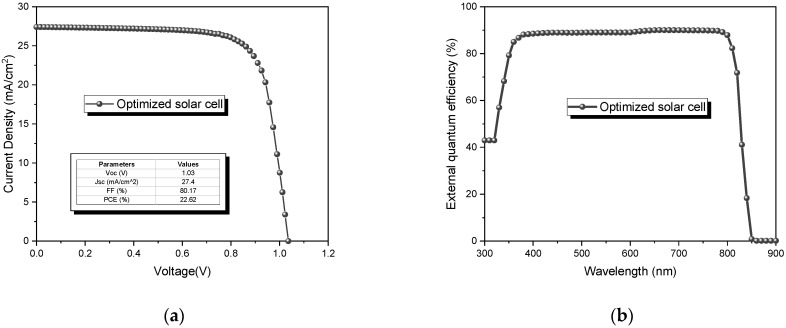
Optimized CdTe solar cell: (**a**) J-V curve and (**b**) external quantum efficiency.

As the last step, a new simulation was carried out considering the optimized characteristics obtained in the previous sections, such as thicknesses of 3 µm for CdTe and 25 nm for CdS, a work function of 5.7 eV, a defect density of CdTe of 10^13^ cm^−3^, a negligible series resistance, a shunt resistance of 3000 Ω cm^2^, and hole carrier concentration of 10^17^ cm^−3^. In addition, the use of ZnO:Al and CuSCN layers was considered. The J-V curve and EQE are presented in Figure 12. The solar cell performance is shown to be improved, as expected. An efficiency of 22.62% was obtained, slightly superior to the solar cell record (22.1%) [60]. Furthermore, an increase in the EQE at lower wavelengths was observed, where cell 1 cannot absorb.

## 4. Conclusions

The understanding of CdTe solar cell physics is valuable for achieving greater efficiencies. In this sense, a numerical simulation (SCAPS) of the CdTe device was carried out. The incorporation of the ZnO:Al nanolayer (TCO) and CuSCN nanolayer (HTL) on solar cell performance was compared and studied. The device configurations were: cell 1, CdTe/CdS/SnO_2_:F; cell 2, CdTe/CdS/ZnO:Al; and cell 3, CuSCN/CdTe/CdS/ZnO:Al. First, the influence of different parameters (thickness, work function, bulk defects, carrier concentration, and Rs) on cell 1 was studied and explained. Then, the impact of the ZnO:Al and CuSCN was evaluated for cells 2 and 3, respectively. Promising optimized results were achieved with a conversion efficiency from 16.04% (cell 1) to 22.62% (cell 3). Finally, cell 3 demonstrated that incorporating ZnO:Al and CuSCN made it possible to obtain a superior V_oc_ and, consequently, greater efficiency. Furthermore, the third device showed a lower adverse effect against parasitic resistance (Rs). This new proposal will guide the feasible fabrication of higher-efficiency CdTe-based photovoltaic cells.

## Figures and Tables

**Table 1:** Input parameters for the simulation of CdTe solar cell.

Properties	CuSCN	CdTe	CdS	ZnO:Al	SnO_2_:F
${µ}_{m}$	0.030	4.0	0.025	0.5	0.5
Eg (eV)	3.4	1.45	2.4	3.3	3.6
χ (eV)	1.9	3.9	4.0	4.0	4.0
ℇr	10	9.4	10	9.0	9.0
$N_{C}$ (${cm}^{-3}$)	1.7 × 10^19^	8 × 10^17^	2.2 × 10^18^	1 × 10^19^	2.2 × 10^18^
$N_{V}$ (${cm}^{-3}$)	2.5 × 10^21^	1.8 × 10^19^	1.8 × 10^19^	1 × 10^19^	1.8 × 10^19^
${µ}_{n}$ (cm2VS)	1 × 10^−4^	3.2 × 10^2^	1.0 × 10^2^	5	1.0 × 10^2^
${µ}_{p}$ (cm2VS)	1 × 10^−1^	4	2.5	5	2.5
$N_{d}\;({cm}^{-3})$	-	-	1.1 × 10^18^	5 × 10^17^	1.1 × 10^17^
$N_{a}\;({cm}^{-3})$	1 × 10^18^	2 × 10^14^	-	-	-
$N_{t}\;({cm}^{-3})$	1 × 10^14^	2 × 10^14^	1 × 10^18^	1 × 10^15^	1 × 10^15^
Defect type	Single donor	Single donor	Single acceptor	Single acceptor	Single acceptor

**Table 2:** CdTe solar cell parameters (CdS/CdTe interface).

CdS/CdTe Interface	Values
Defect type	acceptor
Capture cross-section for electrons (cm^2^)	1 × 10^−13^
Capture cross-section for holes (cm^2^)	1 × 10^−13^
Energetic distribution	Single
Reference for defect energy level Et	Above Ev of CdTe
Energy concerning reference (eV)	0.100
Total density (integrated over all energies) (1 cm^2^) at CdS/CdTe interface	1.6 × 10^12^

**Table 3:** Effect of the CdTe thickness on the output parameters.

Output Parameters	500 nm	1000 nm	1500 nm	2000 nm	2500 nm	3000 nm	3500 nm	4000 nm	4500 nm	5000 nm
V_oc_ (V)	0.69	0.73	0.79	0.83	0.85	0.85	0.86	0.87	0.87	0.87
J_sc_ (mA/cm^2^)	20.92	23.04	23.63	23.89	24.03	24.11	24.15	24.18	24.19	24.20
FF (%)	81.57	81.23	79.37	78.94	78.53	77.82	77.01	76.14	75.29	74.45
PCE (%)	11.83	13.78	14.95	15.73	16.05	16.13	16.11	16.04	15.93	15.80

**Table 4:** Effect of the CdS thickness on the output parameters.

Output Parameters	50 nm	15 nm	20 nm	25 nm	30 nm	35 nm	40 nm	45 nm	50 nm
V_oc_ (V)	0.87	0.87	0.87	0.87	0.87	0.87	0.87	0.86	0.86
J_sc_ (mA/cm^2^)	24.89	24.63	24.40	24.18	23.97	23.77	23.58	23.40	23.22
FF (%)	76.18	76.17	76.15	76.14	76.13	76.12	76.11	76.10	76.09
PCE (%)	16.53	16.35	16.19	16.04	15.89	15.75	15.62	15.49	15.37

**Table 5:** J-V curves of different CdTe solar cell configurations.

Output Parameters	CdTe/CdS/SnO_2_:F	CdTe/CdS/ZnO:Al	CuSCN/CdTe/CdS/ZnO:Al
V_oc_ (V)	0.87	0.87	0.94
J_sc_ (mA/cm^2^)	24.18	25.5	24.91
FF (%)	76.14	76.19	75.67
PCE (%)	16.04	16.90	17.72

## Data Availability

Not applicable.
